# Oxidative stress-induced stress granules: a central link to protein aggregation in neurodegenerative diseases

**DOI:** 10.3389/fnins.2025.1686571

**Published:** 2025-11-07

**Authors:** Neelam Younas, Inga Zerr

**Affiliations:** 1 Department of Neurology, National Reference Center for Surveillance of TSE, University Medical Center Göttingen, Göttingen, Germany; 2 German Center for Neurodegenerative Diseases (DZNE), University of Göttingen, Göttingen, Germany

**Keywords:** oxidative stress, stress granules, pathological aggregation, persistent SGs, tauopathies

## Abstract

Intracellular aggregation of proteins such as Tau, TDP43, FUS, prion protein, and α-synuclein is a major hallmark of many major neurodegenerative diseases. Aberrant stress granules (SGs) are emerging as key contributors to the nucleation of toxic protein aggregates in these disorders. SGs are dynamic, membrane less cytoplasmic assemblies that form transiently through liquid–liquid phase separation (LLPS) of RNA binding proteins (RBPs) containing low complexity domains, together with stalled mRNAs, to help cells cope with stress. While physiological SGs facilitate cellular resilience to acute stress and undergo rapid disassembly, chronic or excessive stress leads to persistent SGs, driving pathological protein aggregation characteristic of age related neurodegeneration. The inherent reversible aggregation of RBPs crucial for cellular function paradoxically exposes them to misfolding disorders. Notably, recent findings expand this paradigm by demonstrating that Tau itself participates in SG formation, with Tau–SG interactions potentiating Tau aggregation and disease progression in tauopathies. Despite these insights, the precise cellular stressors and posttranslational modifications (PTMs) governing the shift from physiological granules to pathological aggregates remain poorly defined. Emerging evidence highlights oxidative stress as a central upstream mediator of this transition. In this perspective, we synthesize current understanding of how SG dynamics intersect with oxidative stress to potentiate protein aggregation, proposing molecular mechanisms that bridge SG biology and neurodegenerative disease. Elucidating these pathways is essential for the development of targeted therapeutic interventions for disorders such as Alzheimer’s disease and related tauopathies.

## 1 Introduction

A key hallmark of major neurodegenerative disorders—including Alzheimer’s disease (AD), amyotrophic lateral sclerosis (ALS), frontotemporal dementia (FTD), synucleinopathies and prion diseases—is the intracellular accumulation of aggregated proteins such as MAPT, TDP43, FUS, PRNP and SNCA (Wilson et al., 2023).

Although significant progress has been made in delineating the aggregation process, the exact cellular mechanisms that precipitate pathological aggregation remain inadequately resolved.

**FIGURE 1:**
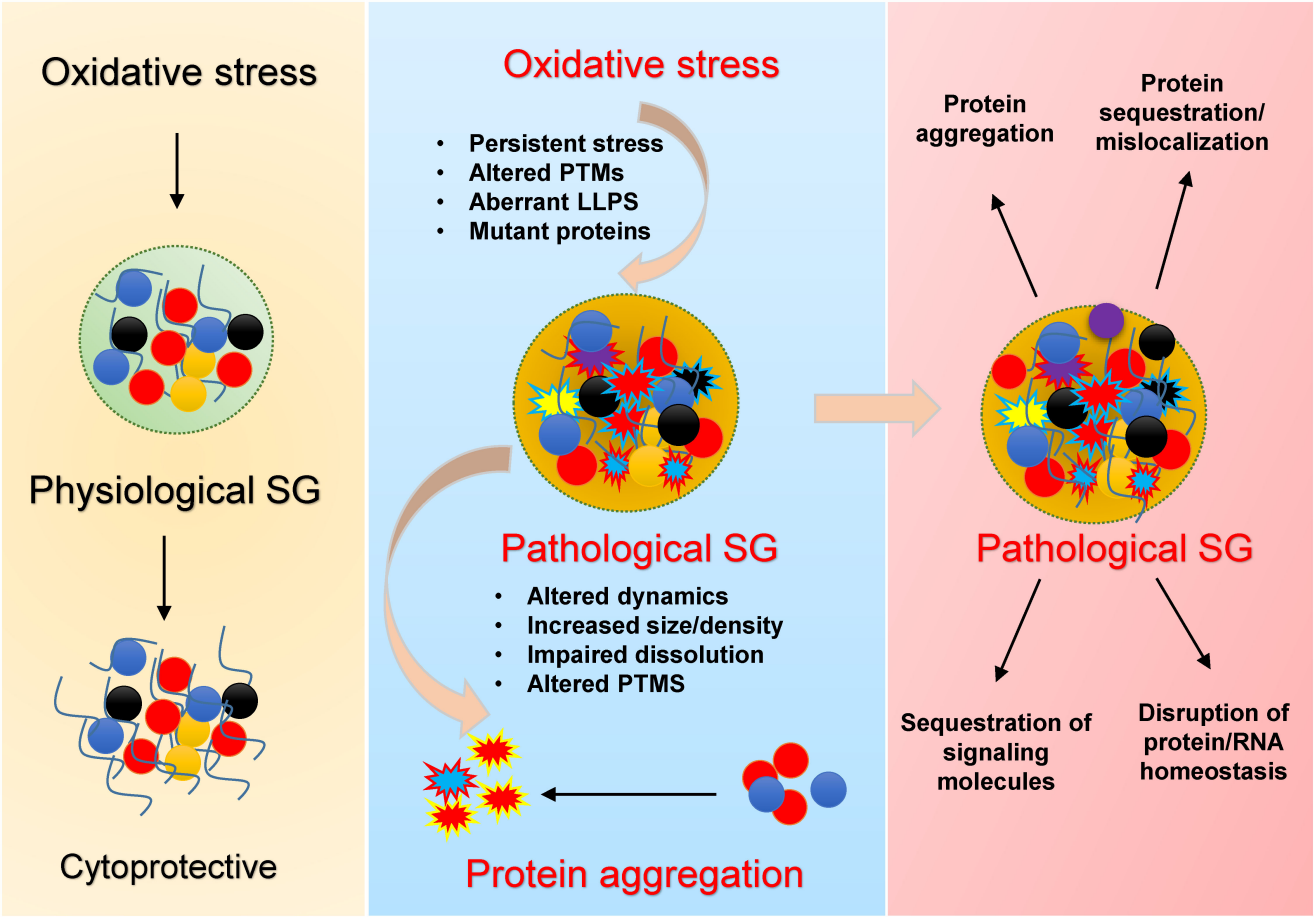
Mechanistic overview by which oxidative stress promotes pathological stress granule (SG) formation and protein aggregation. Under acute stress, SGs transiently assemble, sequester RNAs and proteins, and regulate translation, supporting cell survival during stress (left). Chronic/recurrent oxidative stress promotes persistent SGs, oxidation of key residues, abnormal PTMs, and LLPS transitions, especially in the presence of disease mutations (middle), leading to conversion of dynamic liquid-like SGs into dense, pathological condensates. These aberrant SGs display impaired dissolution, altered composition, and serve as nucleation sites for neurodegeneration-associated protein aggregates. Their persistence (right) drives protein misfolding and mislocalization, sequestration of signaling factors, disruption of protein/RNA homeostasis, and ultimately neuronal dysfunction.

Newly identified elements of these aggregates are stress granules (SGs)–membrane less cytoplasmic assemblies of RNA-binding proteins and stalled RNA translational machinery–as crucibles of aggregation (Anderson and Kedersha, 2008; Fomicheva and Ross, 2021). Stress granule dynamics are conserved throughout eukaryotes and represent a universal cytoprotective mechanism against diverse environmental stresses (Kedersha et al., 2013) including oxidative stress, heat shock, osmotic stress, UV irradiation, and nutrient deprivation (Cabral et al., 2022; Glauninger et al., 2022; Kedersha et al., 1999). Under physiological conditions, SGs are transient, rapidly dissolving upon stress resolution. However, chronic or persistent SGs—often observed with aging or continuous stress exposure—may act as nucleation centers for disease-associated protein aggregation, thereby contributing to neurodegenerative pathology (Figure 1).

Multiple lines of evidence provide a strong evidence that SGs in cells exposed to environmental stress play the role of seeds for pathogenic protein aggregation in neurodegenerative disorders. First, several proteins [MAPT (Maziuk et al., 2018; Vanderweyde et al., 2016; Younas et al., 2020), TDP43 (Yan et al., 2025), FUS, SNCA (Younas et al., 2023b) and PRNP (Younas et al., 2023b)] that mount up in cytoplasmic aggregates are also components of these SGs (Ash et al., 2021; LeBlang et al., 2020; Yan et al., 2025; Younas et al., 2023b). Second, long lifespan and high metabolism of neurons make them specifically vulnerable to successive stress episodes and to cycles of SG assembly-disassembly (Bishop et al., 2010). Third, mutations in the disease-linked proteins can disrupt normal dynamics of SGs (Lenzi et al., 2015; Mateju et al., 2017; Murakami et al., 2015). Additionally, mutations in other components of SGs e.g., hnRNPA1 (Clarke et al., 2021), TIA1 (Apicco et al., 2018; Baradaran-Heravi et al., 2020; Jiang et al., 2022), ATXN2 (Guevara-García et al., 2012; Kato et al., 2019), UBQLN2 (Peng et al., 2022; Yan et al., 2025) can also disrupt SG dynamics (Table 1). Fourth, SG dense cores act as hotspots for protein aggregation (Maziuk et al., 2018; Vanderweyde et al., 2016; Yan et al., 2025). Stress granules (SGs) have emerged as a critical missing link that integrates genetic, cellular, and pathological evidence, completing the puzzle of aberrant protein aggregation cascade in neurodegenerative diseases (Cui et al., 2024; Dudman and Qi, 2020; Gu et al., 2024; Yuan et al., 2025). A summary of key proteins, their associated stressors, reported mutations, models used for study, post-translational modifications (PTM), and supporting references are described in Table 1.

**TABLE 1:** Summary of different RBPs or non-RBPs linked with specifically oxidative stress-induced stress granule pathology in neurodegenerative diseases.

Protein	Stressor	Mutations	Model	PTM status	References
MAPT	Sodium arsenite, pathophysiological stress	P301L, P301S, G272V	Cell lines, mouse brain, human brain (endogenous)	Hyperphosphorylation, truncation, acetylation	Maziuk et al., 2018; Vanderweyde et al., 2016; Younas et al., 2020; Kanaan et al., 2020; Shelkovnikova et al., 2017; Vanderweyde et al., 2012
SFPQ	Sodium arsenite, pathophysiological stress	N533H, L534I	Cell lines, live cells, human brain	Oxidation, Phosphorylation	Younas et al., 2020; Ke et al., 2012; Lukong et al., 2009; Neeves et al., 2025; Uechi et al., 2025; Widagdo et al., 2022
TDP43	Sodium arsenite, heat shock, sorbitol or paraquat, pathophysiological stress	G294A, A315T, Q331K, Q343R, G348C, M337V, R361S	Cell lines, primary glia, human brain or spinal cord	Oxidation, Phosphorylation, S-nitrosylation	Dewey et al., 2011; Liu-Yesucevitz et al., 2011; McDonald et al., 2011; McGurk et al., 2014; Parker et al., 2012
FUS	Sodium arsenite, traumatic injury or heat shock	R521C, R495X, H517Q, P525L	Mouse primary motor neuron, mouse spinal cord, drosophila brain or zebrafish spinal cord	Methylation, phosphorylation, acetylation	Baron et al., 2013; Bosco et al., 2010; Zhang et al., 2020
PRNP	Sodium arsenite, pathophysiological stress	–	Cell lines, mouse brain,	Phosphorylation, glycosylation, truncation	Wolschner et al., 2009; Younas et al., 2023b; Yun et al., 2006
SNCA	Sodium arsenite, ↑OS interacts with mutations (H50Q, E46K), αS toxicity linked to P-bodies	H50Q, E46K, A53T, A30P	Cell lines, yeast, Drosophila, and human genetics	Phosphorylation (S129), nitration, sumoylation	Hallacli et al., 2022; Younas et al., 2023b
hnRNPA1	Sodium arsenite, heat shock, or sorbitol, pathophysiological stress, OS induces stress granule pathology	D290A, P288A, D262V, *321Eext*6, *321Qext*6, and G304Nfs*3	Cell lines, human fibroblast cells from ALS/FTD patients	Phosphorylation, PAR-ylation	Beijer et al., 2021; Duan et al., 2019; Guil et al., 2006; Kim et al., 2013
TIA1	Sodium arsenite, pathophysiological stress	P362L	Cell lines, mouse brain, human brain	Oxidation, PAR-ylation	Maziuk et al., 2018; Vanderweyde et al., 2016; Younas et al., 2020; Arimoto-Matsuzaki et al., 2016; Mackenzie et al., 2017; Shelkovnikova et al., 2017; Vanderweyde et al., 2012
ATXN2	Sodium arsenite	Toxic polyQ expansions	Yeast cells, mouse (SCA2), cellular models	Oxidation, phosphorylation, LLPS interactions	Guevara-García et al., 2012; Kato et al., 2019
UBQLN2	OS increases stress granules, aggregation	P497H, P497S, A488T	iPSC-motor neurons, KO mice	Phosphorylation, aggregation, altered LLPS	Gu et al., 2024; Peng et al., 2022

OS, oxidative stress; LLPS, liquid-liquid phase separation; αS, alpha synuclein.

## 2 Oxidative stress-induced stress granules as key drivers of pathological aggregation

A central unresolved question in the field is whether specific stressors preferentially drive the conversion of physiological protein condensates into pathogenic aggregates *in vivo*, especially under prolonged or recurrent stress. A growing body of research points toward oxidative stress as a principal upstream trigger in this pathogenic cascade (Gu et al., 2024; Ratti et al., 2020). Recent work by Yan et al. (2025) demonstrates that the aggregation of TDP-43 within SGs not only requires high protein concentrations but also strictly depends on oxidative insults. Oxidative agents such as arsenite and paraquat oxidize cysteine residues, which is crucial for induction of TDP-43 demixing within condensates, finally converting it into pathological aggregates. By contrast, stress granules induced by non-oxidative agents (e.g., puromycin) fail to trigger aggregation—even at high TDP-43 levels and with impaired proteasomal or chaperone systems—unless oxidative stress is also present (Yan et al., 2025).

Similar mechanisms appear operative with Tau protein, the signature protein in AD and related tauopathies (Younas et al., 2020). Oxidative stress (induced by sodium arsenite) lead to the upregulation of TIA-1 (a classical marker of SGs) and phosphorylation of Tau in cellular models in both neuronal and non-neuronal cell lines, and human brain of AD cases (Alavi Naini and Soussi-Yanicostas, 2015; Younas et al., 2020). Tau phosphorylation–a hallmark of AD–promotes its phase separation and aggregation *in vitro* (Ambadipudi et al., 2017). Chronic or repeated oxidative stress, coupled with elevated levels of TIA-1 and phosphorylated Tau, may create a microenvironment within SGs that nucleates pathological aggregation. Thus, stress granules may represent the missing mechanistic link bridging oxidative damage, protein modification, and aggregate formation in neurodegenerative diseases (Figure 1).

Outstanding mechanistic questions remain, especially regarding which molecular species of Tau participate in SG formation. Are they distinct from the Tau species involved in the stabilization of microtubules? Previously it has been reported that a shift in localization of Tau toward somatodendritic compartments occurs to facilitate formation of SGs (Vanderweyde et al., 2016). However, it is unlikely that the same Tau species involved in microtubule stabilization are also responsible for stress granule formation, as this would compromise microtubules integrity during stress. It is more plausible that nuclear Tau species (Younas et al., 2023a) might be involved in the formation of SGs. Tau has six isoforms and many posttranslational modifications. Post-translational modifications also affect stress granules dynamics (Wang et al., 2023). Certain pathological Tau species—especially those that are hyperphosphorylated—show enhanced association with SGs, impacting SG dynamics and contributing to neurodegenerative mechanisms. This mechanistic link underscores the importance of isoform diversity and PTMs in how Tau modulates SG formation and pathological aggregation. Further research is needed to clarify this important issue. By focusing future research on the molecular interactions between oxidative stress, SG dynamics, and protein aggregation, this emerging concept has the potential to transform our strategies for early diagnosis, disease monitoring, and the development of novel treatments for Alzheimer’s disease and related tauopathies.

Chronic and recurrent oxidative stress in the context of mutant proteins—whether RNA binding or not—can drive the formation of pathological SGs (Table 1). Notably, mutations in SG-associated genes—including MAPT, TARDBP (TDP-43), FUS, ATXN2, TIA1, and HNRNPA1—have been shown to disrupt SG dynamics, impair disassembly, and enhance aggregation propensity, thereby contributing to neurodegenerative disease pathogenesis (Table 1).

This is also important to note that the vast majority of knowledge about oxidative stress and stress granule dynamics is derived from experimental exposure to artificial oxidants (e.g., sodium arsenite, H2O2) in cellular models. There is limited direct evidence demonstrating that physiologically relevant endogenous reactive oxygen species levels induce the same oxidative modifications and phase behaviors under disease conditions *in vivo* (Table 1). The conversion of protective, metastable condensates into pathogenic aggregates within stress granules is determined by a complex interplay of the protein involved, nature of oxidative insult, and the posttranslational landscape.

Taken together, these findings suggest that stress granules could represent a missing mechanistic link connecting oxidative damage, protein modification, and aggregate formation in neurodegenerative diseases (Figure 1). This mechanistic specificity underscores the potential for developing targeted, redox-oriented neuroprotective therapies aimed at modulating SG composition and stability under oxidative conditions. Advancement in this area will require deeper investigations into physiologically relevant stress profiles, diversity of isoforms and posttranslational modifications, and validation in robust *in vivo* models. These efforts hold promise for translating mechanistic insights into tangible clinical interventions that halt or reverse the aggregation process.

## 3 Conclusion

In conclusion, converging evidence positions stress granules as a mechanistic nexus where oxidative stress and altered protein homeostasis intersect to drive pathological aggregation in neurodegenerative disorders. The conversion of metastable, protective condensates into pathogenic aggregates appears to be highly contingent on the nature and duration of cellular insults—particularly oxidative stress—rather than on SG formation *per se*. This duality underscores the therapeutic potential of targeting stress granule dynamics and redox homeostasis, but also highlights the need for more granular understanding of the molecular players and contextual triggers involved. As our mechanistic insights deepen, SGs can offer a promising frontier for both biomarker discovery and intervention in proteinopathic neurodegeneration.

## Data Availability

The original contributions presented in this study are included in this article, further inquiries can be directed to the corresponding author.
